# Diurnal variation of phenylalanine and tyrosine concentrations in adult patients with phenylketonuria: subcutaneous microdialysis is no adequate tool for the determination of amino acid concentrations

**DOI:** 10.1186/1475-2891-12-60

**Published:** 2013-05-14

**Authors:** Sarah C Grünert, Corinna M Brichta, Andreas Krebs, Hans-Willi Clement, Reinhold Rauh, Christian Fleischhaker, Klaus Hennighausen, Jörn Oliver Sass, K Otfried Schwab

**Affiliations:** 1 Center for Pediatrics and Adolescent Medicine, University Hospital Freiburg, Freiburg, Germany; 2 Department of Child and Adolescent Psychiatry and Psychotherapy, University Hospital Freiburg, Freiburg, Germany; 3 Clinical Chemistry and Biochemistry, University Children’s Hospital Zürich, Zürich, Switzerland

**Keywords:** Phenylketonuria, Phenylalanine, Tyrosine, Diurnal variation, Diet, Physical exercise, Microdialysis, Dried blood spot, Ergometry

## Abstract

**Background:**

Metabolic control and dietary management of patients with phenylketonuria (PKU) are based on single blood samples obtained at variable intervals. Sampling conditions are often not well-specified and intermittent variation of phenylalanine concentrations between two measurements remains unknown. We determined phenylalanine and tyrosine concentrations in blood over 24 hours. Additionally, the impact of food intake and physical exercise on phenylalanine and tyrosine concentrations was examined. Subcutaneous microdialysis was evaluated as a tool for monitoring phenylalanine and tyrosine concentrations in PKU patients.

**Methods:**

Phenylalanine and tyrosine concentrations of eight adult patients with PKU were determined at 60 minute intervals in serum, dried blood and subcutaneous microdialysate and additionally every 30 minutes postprandially in subcutaneous microdialysate. During the study period of 24 hours individually tailored meals with defined phenylalanine and tyrosine contents were served at fixed times and 20 min bicycle-ergometry was performed.

**Results:**

Serum phenylalanine concentrations showed only minor variations while tyrosine concentrations varied significantly more over the 24-hour period. Food intake within the patients’ individual diet had no consistent effect on the mean phenylalanine concentration but the tyrosine concentration increased up to 300% individually. Mean phenylalanine concentration remained stable after short-term bicycle-exercise whereas mean tyrosine concentration declined significantly. Phenylalanine and tyrosine concentrations in dried blood were significantly lower than serum concentrations. No close correlation has been found between serum and microdialysis fluid for phenylalanine and tyrosine concentrations.

**Conclusions:**

Slight diurnal variation of phenylalanine concentrations in serum implicates that a single blood sample does reliably reflect the metabolic control in this group of adult patients**.** Phenylalanine concentrations determined by subcutaneous microdialysis do not correlate with the patients’ phenylalanine concentrations in serum/blood.

## Background

Phenylketonuria (PKU, OMIM 261600) is an autosomal recessive disorder of phenylalanine (Phe) metabolism caused by mutations in the *PAH* gene [1]. The enzyme phenylalanine hydroxylase (PAH, EC 1.14.16.1), a cytosolic homotetramer, catalyzes the hydroxylation of Phe to tyrosine (Tyr) using tetrahydrobiopterin (BH_4_) as a cofactor [1]. *PAH* gene mutations may result in variable degrees of enzyme deficiency [1]. In untreated PKU patients, the accumulation of Phe and a relative deficiency in Tyr lead to neurological and cognitive impairment of variable degree [1,2]. If initiated early, dietary treatment by a Phe-restrictive and Tyr-supplemented diet can prevent mental retardation and allows for normal intellectual development [3], although several studies have demonstrated subtle impairment of cognitive function in affected patients [4]. Current guidelines recommend regular monitoring of Phe levels by blood sampling at age-adapted time intervals [3,5-7]. However, conditions of blood sampling, such as the time of day, fasting or non-fasting etc. are often not well-specified, and intermittent variations of Phe levels between two measurements remain unknown. Information on daytime variations of Phe and Tyr concentrations and the influence of meals and physical exercise are sparse. Several studies with small groups of PKU patients – mainly children - have been performed [8-13], yielding controversial results. Although the long-term outcome of treated PKU patients over the last decades suggests that diurnal variations of phenylalanine and tyrosine concentrations are small in comparison with day to day variability and do not have a major impact on the course of the disease, they are certainly of academic interest.

Subcutaneous microdialysis (MD) allows continuous collection of samples from the interstitial space fluid of subcutaneous adipose tissue. An advantage of this technique is the possibility of acquiring a series of diagnostic samples without single blood drawings [14]. Although the method has proved useful to determine in vivo tissue concentrations of amino acids in healthy individuals [15,16], it has not been studied in patients with PKU yet.

In MD a small semipermeable hollow fiber dialysis catheter is inserted into the tissue [17]. A pump continuously perfuses this catheter with a solution (perfusate) at very slow rates (microlitres per min). Within the MD catheter, the equilibrium with the environment (extracellular space of the tissue) is achieved by means of diffusion of analytes from the interstitial fluid into the perfusate through the semipermeable membrane [18]. Diffusion through the membrane is limited to soluble substances of low molecular weight [19].

This study addresses the question whether subcutaneous MD may be helpful in the assessment of Phe and Tyr serum concentrations. Therefore, amino acid concentrations in MD fluid were compared to the concentrations in serum and dried blood of adult PKU patients. Another aim of this study was to elucidate if in adult PKU patients a single blood sample can reliably reflect the overall metabolic control and to examine to what extent the oral Phe intake (within the patients’ individual dietary limits) and physical exercise influence Phe and Tyr concentrations.

## Methods

### Patients

Eight adults with PKU (Phe concentrations in serum > 600 μmol/l, if untreated) were included in this study. Age ranged between 20 and 44 years (median 31 years). Details on age, sex, *PAH* mutations, daily Phe and Tyr intake and daily amino acid supplements are displayed in Table 1. All but one patient followed a Phe-restricted diet. Patients with acute or chronic diseases other than PKU, neuropsychiatric diseases, pregnant patients and patients on regular medication other than oral contraceptives or amino acid supplements were excluded from this study.

**Table 1:** Study population of 8 PKU patients

**Patient**	**Age (years)**	**Sex**	***PAH *gene mutations nucleotide change (Amino acid change)**	Phe intake (mg/24 h)*	Phe intake (mg/kg/24 h)*	**Tyr intake (mg/24 h)#**	**Tyr intake (mg/kg/24 h)#**	Amino acid supplements (g protein/24 h)** (SD)##	Amino acid supplements (g protein/kg/24 h)**
						**Total from food from amino acid supplements**			
1	42	m	c.842C>T	1071	14	9057	115	80 (3)	1,0
			(p.P281L)						
						6080			
			c.842C>T						
						2977			
			(p.P281L)						
2	20	f	n.a.	1653	35	1144	24	0 (0)	0,0
						0			
						1140			
3	22	f	c.842C>T	1142	12	6191	66	61 (3)	0,6
			(p.P281L)						
						3660			
			c.842C>T						
						2531			
			(p.P281L)						
4	32	m	c.580_581delCT	1083	13	5316	63	51 (3)	0,6
			(p.L194Efs*5)			3060			
						2256			
			c. 782G>A						
			(p.R261Q)						
5	32	f	c.473G>A	1395	19	5045	69	45 (3)	0,6
			(p.R158Q)						
						2700			
			c.473G>A						
						2345			
			(p.R158Q)						
6	26	f	c.1222C>T	1149	18	1378	21	0 (0)	0,0
			(p.R408W)			0			
			c.1066-3C>T						
						1378			
			(p.?)						
7	28	f	c.782G>A	1090	13	8329	97	71 (3)	0,8
			(p.R261Q)			4757			
			c.1222C>T			3572			
			(p.R408W)						
8	44	f	c.60+5G>T	1556	26	4084	69	31 (3)	0,5
			(p.?)						
						1266			
			c.727C>T						
						2818			
			(p.R243*)						

* Phenylalanine intake (mg) during study visit calculated on basis of dietary protocols.

# Tyrosine intake (mg) during study visit calculated on basis of dietary protocols specified as total intake, tyrosine intake from food and tyrosine intake from amino acid supplements.

** Intake of amino acid supplements (g protein) during study visit.

## Distribution of amino acids supplements during study visit in number of single doses (SD).

m = male; f = female.

Before inclusion in the study physical examination was performed. Patients were examined during a 26-hour study visit at the University Hospital in Freiburg, Germany. Informed written consent was obtained from all study participants. Our study was approved by the Ethics Committee of the University of Freiburg, Germany (149/10).

### Schedule of the study visit

**Figure 1:**
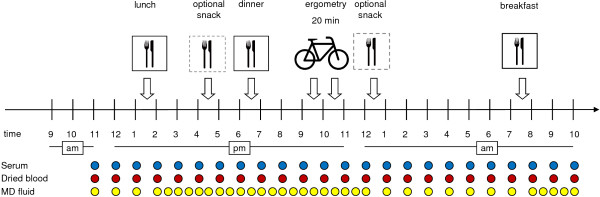
**Schedule of the study visit.** Schedule of the study visit with meals and optional snacks at fixed times and bicycle ergometry at either 9.15 pm or 10.15 pm. The sequence of drawing blood samples (for serum and dried blood specimens) and the sequence of sampling microdialysis fluid (MD fluid) are displayed as coloured dots.

The schedule of the study visits is displayed in Figure 1. Each study visit started at 9 am. Within the first two hours a peripheral venous catheter was inserted and a MD catheter was introduced into the periumbilical subcutaneous adipose tissue. From 11 am on, serum and dried blood samples were collected hourly. MD fluid samples were also collected every hour and additionally every 30 minutes between 2 and 12 pm and 8 and 10 am to capture possible postprandial changes in amino acid concentrations. During the study all patients continued their regular individual diet including amino acid supplements (Table 1). A diet protocol was written before the study visit and the meals were prepared according to this protocol during the study visit. Three meals with defined Phe and Tyr contents were served at fixed times (Figure 1). In addition, two snacks with a maximum Phe content of 190 mg were offered. Any remaining food was reweighed and deducted from the total start weight. The exact phenylalanine and tyrosine contents of all meals were calculated by a dietician.

20 minutes of physical exercise by cycle ergometry was perfomed between 9 and 11 pm. Ergometry was initiated with 50 W and successively adapted (50 W increase every two minutes) to patients’ heart rate to yield similar physical exertion in all patients.

### Microdialysis and sample collection

The concentration of a particular substance in the perfusate relative to its concentration in the surrounding medium (interstitial fluid) is called “relative recovery“ [17]. MD studies to evaluate the determination of glucose in subcutaneous adipose tissue indicated that slow flow rates (0.3-0.5 μmol/min) in combination with large MD probes (30 mm membrane) allow almost complete recovery of low molecular mass substances [20,21]. We used a CMA 63 microdialysis catheter (CMA/Microdialysis, Solna, Sweden) with a membrane length of 30 mm and a diameter of 0.6 mm. The molecular exclusion size of the polyamide membrane was 20 kD. The catheter was perfused by a CMA 107 microdialysis pump at a perfusion rate of 0.5 μl/min with Ringer solution, samples were taken hourly and postprandially half-hourly (Figure 1). 7.1 μl microdialysate of every sample were pipetted on special filter paper (Whatman [previously Schleicher & Schuell] 903, obtained from GE Healthcare) and dried at room temperature. Blood for amino acid determination was collected in serum tubes (S-Monovette 9 ml, Sarstedt, Nümbrecht, Germany) and centrifuged after coagulation (after 30 min). Serum samples were stored in Eppendorf cups at -80°C until analysis. One drop of freshly drawn whole blood was applied to filter paper cards (see above) by the same person during all study visits ensuring a comparable quality of dried blood samples. Filter paper cards were dried at room temperature before analysis.

### Assessment of amino acid concentrations in serum, dried blood spots and MD fluid

Phe and Tyr concentrations in dried blood spots and dried microdialysate spots on filter paper were analysed using tandemmass spectrometry (2795 Alliance HT (HPLC), Quattro micro (tandem mass detector), Waters GmbH, Eschborn, Germany). For internal quality control reference filterspots with defined amino acid concentrations (ClinChek® Whole Blood Control (filterspot) for Acylcarnitines and Amino Acids, Level I and Level II, Lot. 105, Recipe Chemicals + Instruments GmbH, Munich, Germany) were used and measured together with the patients’ samples. Results were considered reliable only if both controls yielded concentrations within the reference range provided by the manufacturer. Quality of dried blood spot amino acid analysis was also ensured by participation in the Newborn Screening Quality Assurance Program for laboratory testing, Center for Disease Control and Prevention, Atlanta, Georgia, USA and the Association of Public Health Laboratories, Washington, D.C., USA, and the Quality Assurance Program, LADR GmbH, MVZ Dr. Kramer & Colleagues, Geesthacht, Germany.

Serum amino acids were determined after protein precipitation with sulfosalicylic acid on a Biochrom B30 amino acid analyzer (Biochrom Ltd., Cambridge, England) using ion-exchange chromatography followed by post-column derivatization with ninhydrin. External control was performed using the corresponding INSTAND quality control scheme (INSTAND e.V., WHO Collaborating Centre for the Quality Assurance and Standardization in Laboratory Medicine, Düsseldorf, Germany).

### Statistical analysis

Statistical analysis was performed using IBM SPSS Statistics 20. Before applying parametric tests, dependent variables were tested for deviations from normal distribution by the Shapiro-Wilk test (*p > .05* in 93% of the variables). Wilcoxon signed-rank test was used to compare variation coefficients of Phe and Tyr. To evaluate the short-term effect of meals and exercise on Phe and Tyr concentrations repeated-measures MANOVA were performed with the within-subjects factor “time of measurement” (before the meal, 60 min after the meal, 120 min after the meal). To assess the correlation of Phe and Tyr concentrations between different kinds of samples (serum, dried blood and microdialysate) Pearson’s correlation coefficients (*r*) were calculated for each patient, then transformed to Fisher-*Z* values (Fisher r-to-Z transformation) and averaged. Resulting mean Fisher-Z values were retransformed to *r* values (reversed Fisher transformation).

## Results

### Serum Phe and Tyr concentrations

**Figure 2:**
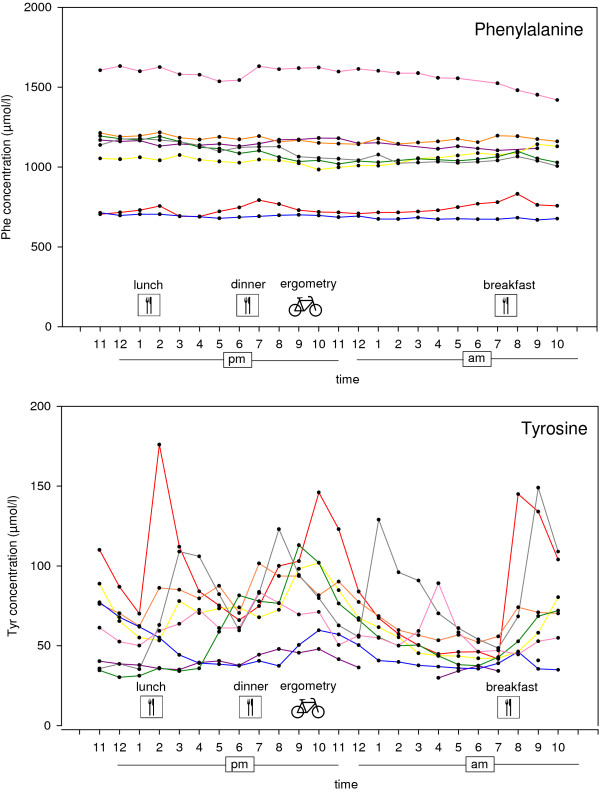
**Variation of phenylalanine and tyrosine concentrations in serum over 24 hours.** Profiles of phenylalanine (Phe) and tyrosine (Tyr) concentrations in serum of all patients during the 24-hour study visit. Time of main meals and bicycle ergometry is displayed.

Mean Phe concentration in serum ranged between 687 and 1573 μmol/l. Mean Tyr concentration was between 39 and 89 μmol/l. There was very small variation in Phe concentrations over the time studied (Figure 2). The variation coefficient for Phe was 3% ([95% CI: 2.5–4.8], whereas Tyr concentrations showed significantly greater variation (variation coefficient 28% [95% CI: 19.3–36.2]; *z = -2.*52, p = .01 (Wilcoxon signed-rank test). The mean variation in Phe concentrations in serum was 132 μmol/l (45 to 212 μmol/l). The mean variation in Tyr concentrations was 68 μmol/l (18 to 134 μmol/l). The highest Phe concentration in most patients was measured between 8 and 12 am. Almost all patients had their lowest Tyr concentration before noon.

### Short-term effect of meals on Phe and Tyr concentrations

The Phe consumption during the meals ranged between 172 and 725 mg per meal according to the patients` individual diet. Seven patients consumed amino acid supplements (daily intake between 7 and 80 g) (Table 1). The statistical analysis of the short-term effect of meals on mean Phe and Tyr concentrations and the Phe:Tyr concentration ratio ([Phe]/[Tyr]) is displayed in Table 2. Preceding dietary intake lead to a significant change (initial rise at 60 min followed by a decline at 120 min) of mean Phe concentration in serum after breakfast (*p* = .038) and lunch (*p* = .023) while no significant effect was observed after dinner. Mean Tyr concentration did not show any significant change after dietary intake while mean [Phe]/[Tyr] declined significantly after breakfast (*p* = .044) and lunch (*p* = .020).

**Table 2:** Short-term effect of meals on mean phenylalanine and tyrosine concentrations and the phenylalanine: tyrosine concentration ratio in serum

	**Before meal**		**60 min postprandial**		**120 min postprandial**			
** Variable**	***M***	***SD***	***M***	***SD***	***M***	***SD***	***F*(2,6**^**a**^**)**	***p***
Phe								
Breakfast	1053	278	1071	257	1040	262	6.74	.04
Lunch	1098	286	1109	288	1082	288	7.59	.02
Dinner	1091	282	1078	280	1059	288	2.60	.15
Tyr								
Breakfast	46	6	70	35	84	42	2.54	.17
Lunch	50	14	74	45	69	31	1.89	.23
Dinner	72	20	82	28	84	25	1.59	.28
[Phe]/[Tyr]								
Breakfast	23	5	18	8	15	7	6.19	.04
Lunch	24	10	19	10	19	10	8.01	.02
Dinner	16	5	15	5	14	6	1.36	.32

Phe = phenylalanine; Tyr = tyrosine; [Phe]/[Tyr] = phenylalanine: tyrosine concentration ratio.

^a^Except for breakfast: n = 7 and therefore denominator df = 5.

To evaluate the short-term effect of meals on phenylalanine and tyrosine concentrations and the phenylalanine: tyrosine concentration ratio in serum repeated-measures MANOVA were performed with the within-subjects factor “time of measurement” (before the meal, 60 min after the meal, 120 min after the meal).

### Short-term effect of exercise on Phe and Tyr concentrations

The results of the statistical analysis of the short-term effect of exercise on mean Phe and Tyr concentrations and [Phe]/[Tyr] are displayed in Table 3. While we could not find any significant effect of short-time exercise on the bicycle ergometer on mean Phe concentration in serum we detected significant effects on Tyr concentration (*p* = .006) and [Phe]/[Tyr] (*p* = .031). Mean Tyr concentration showed a slight rise within one hour after exercise followed by a decline during the following hour. Mean [Phe]/[Tyr] remained stable within the first hour but increased thereafter.

**Table 3:** Short-term effect of exercise on mean phenylalanine and tyrosine concentrations and the phenylalanine: tyrosine concentration ratio in serum

	**Before exercise**		**60 min after exercise**		**120 min after exercise**			
**Variable**	***M***	***SD***	***M***	***SD***	***M***	***SD***	***F*(2,6)**	***p***
Phe	1057	287	1058	291	1050	286	1.46	.30
Tyr	82	25	85	30	69	25	13.31	.01
[Phe]/[Tyr]	14	7	14	7	17	9	6.50	.03

Phe = phenylalanine; Tyr = tyrosine; [Phe]/[Tyr] = phenylalanine: tyrosine concentration ratio.

To evaluate the short-term effect of exercise on phenylalanine and tyrosine concentrations and the phenylalanine:tyrosine concentration ratio in serum repeated-measures MANOVA were performed with the within-subjects factor “time of measurement” (before the exercise, 60 min after the exercise, 120 min after the exercise).

### Comparison of Phe and Tyr concentrations in serum, dried blood and MD fluid

**Figure 3:**
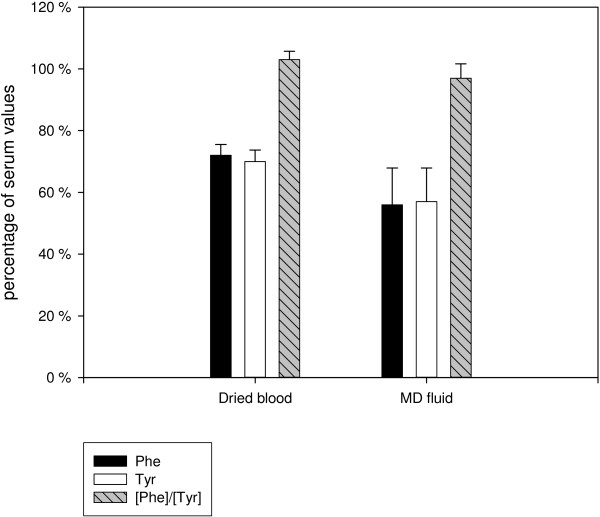
**Phenylalanine and tyrosine concentrations as well as phenylalanine:tyrosine concentration ratio in dried blood spots and MD fluid relative to serum values (100%).** Mean and standard deviation of phenylalanine (Phe) and tyrosine (Tyr) concentrations and of the phenylalanine:tyrosine concentration ratio ([Phe]/[Tyr]) in dried blood and microdialysis fluid (MD fluid) relative to the corresponding serum values (100%).

Amino acid concentrations measured in serum were significantly higher than those measured in dried blood (*p* <.0001) and MD fluid (*p* <.0001). The mean Phe concentration in dried blood was 72% (SD 3%, range 68–76%) of the corresponding Phe concentration in serum. The mean Phe concentration analysed in MD fluid yielded only 56% (SD 12%, range 40–75%) of the serum concentration. Similarly, mean Tyr concentrations in dried blood and MD fluid were also lower compared to Tyr concentration in serum (70%, SD 3%, range 65–75% and mean 57%, SD 12%, range 43-76%, respectively). The mean [Phe]/[Tyr] in dried blood and MD fluid did not differ much from serum values (103%, SD 3%, range 99–106% and 97%, SD 4%, range 91–104%, respectively) (Figure 3).

There was no linear correlation between the Phe concentrations in MD fluid and the corresponding serum concentrations (*r* = -.11, *p* = .64). Correlation analysis for Tyr and [Phe]/[Tyr] resulted in higher r values (*r* = .54, *p* = .007 and *r* = .76, *p* < .001, respectively). Assuming that changes of amino acid concentrations in the subcutaneous compartment may occur with a certain delay, calculations of Pearson’s correlation coefficient conducted with a delay of one and two hours in MD fluid were performed. However, *r* values turned out to be even lower when a possible delay was taken into account (Table 4).

**Table 4:** Correlation of phenylalanine and tyrosine concentrations and phenylalanine: tyrosine concentration ratio between different types of samples

	**Real time**		**Delay 1 h**		**Delay 2 h**	
**Variable**	***r***	***p***	***r***	***p***	***r***	***p***
**Correlation between serum and dried blood**						
Phe	.50	.01				
Tyr	.96	< .001				
[Phe]/[Tyr]	.96	< .001				
**Correlation between serum and microdialysate**						
Phe	-.11	.64	-.15	.48	-.24	.25
Tyr	.54	.01	.23	.28	.04	.84
[Phe]/[Tyr]	.76	< .001	.52	.01	.28	.18

Phe = phenylalanine; Tyr = tyrosine; [Phe]/[Tyr] = phenylalanine: tyrosine concentration ratio.

The correlation analysis of phenylalanine and tyrosine concentrations and of the phenylalanine: tyrosine concentration ratio ([Phe]/[Tyr]) between microdialysis fluid and serum was performed with samples taken simultaneously (“Real time”) and with microdialysate samples taken one hour (“Delay 1 h”) and two hours (“Delay 2 h”) after blood sampling. To assess the above-mentioned correlations Pearson’s correlation coefficients (*r*) were calculated for each patient, then transformed to Fisher-*Z* values (Fisher r-to-Z transformation) and averaged. Resulting mean Fisher-Z values were retransformed to *r* values (reversed Fisher transformation).

Phe concentrations in dried blood and serum showed a moderate correlation (*r* = .50, *p* = .014) whereas again higher correlation coefficients for Tyr (*r* = .96, *p* < .001) and [Phe]/[Tyr] (*r* = .96, *p* < .001) indicated stronger correlation for these parameters (Table 4).

## Discussion

### Serum Phe and Tyr concentrations

Our results indicate that Phe concentrations in adult PKU patients are subject to only very slight diurnal fluctuations implicating that a single blood sample may reliably reflect the Phe-control in this group of patients. Variation seems to be small especially in relation to the daily mean Phe concentration. Several studies to evaluate the variations of Phe concentrations in PKU patients have been performed in the past. Studying a population of nine PKU patients (age 1 to 20 years) van Spronsen et al. also observed only small daily fluctuations of Phe concentrations especially when related to the daily mean Phe concentrations [13]. However, amino acid measurements in this study were limited to the first half of the day (8.30 am -1.30 pm).

In contrast, McDonald et al. studied plasma Phe concentrations in 16 children (1 to 18 years) with PKU over a time period of 24 hours [11]: The median difference between highest and lowest concentrations observed (155 μmol/l/day) did not differ much from the results found in our study cohort (132 μmol/l/day). However, as they studied mainly children under a more Phe-restricted diet, fluctuation of the Phe concentrations in relation to the patients’ daily mean values was much higher compared to our data from adult patients. Ferguson studied twelve young PKU patients (9 to 15 years) under different regimens of Phe intake and distribution of protein substitute and found marked differences of serum Phe profiles even within the same group [9]. Both studies lead to the conclusion that single samples give an incomplete and non-representative indication of Phe-control in many children with PKU.

We could confirm earlier observations by van Spronsen et al. that Tyr concentrations show larger diurnal variations than Phe concentrations [13]. The greater variability could be explained by the high content of Tyr in the amino acid supplements taken by the majority of patients and the relatively small plasma pool, resulting in higher proportional changes.

In accordance with earlier reports, we found maximum Phe concentrations for most patients during the morning hours (8 am - 12 am) [8,10-13]. An overnight rise in Phe concentration is generally thought to be a result of protein catabolism in the fasting state [22]. However, we did not find a linear overnight rise in Phe concentrations in serum as suspected by Farquhar et al. [8]. Nevertheless, as highest Phe and lowest Tyr concentrations were found in the morning, blood sampling for the monitoring of amino acid concentrations in PKU patients should preferably be done at this time of day.

### Short-term effects of meals on Phe and Tyr concentrations

In healthy individuals Phe and Tyr concentrations rise significantly after meals [23]. Fingerhut et al. studied amino acid concentrations in dried blood spots of 92 probands (< 1 to 48 years) and found postprandial increases of Phe and Tyr concentrations of 18% and 14%, respectively [23].

We did not observe a consistent effect of food intake on mean Phe concentration in serum. While after breakfast and lunch a significant rise in Phe levels was observed no such effect became evident after dinner. Notably, in our study setting the patients received their regular diet and the effect of excessive Phe intake was not tested. It needs to be taken into account that mean Phe levels in our patients were much higher than in healthy controls. Therefore, even if the absolute postprandial increase of Phe concentration was the same, the percental change was much lower and thus yielded no statistical significance. In contrast, Tyr concentration increased to more than 300% in individual patients, especially after additional ingestion of amino acid supplements rich in Tyr.

In a study with nine PKU patients van Spronsen et al. also found Phe concentrations to remain rather stable (postprandial increase to 116%) when compared to postprandial Tyr concentrations that were as high as 548% in single patients [13].

In a different study by the same authors plasma Phe responses to different distributions of the daily Phe allowance over the day were tested in seven PKU patients (1 to 20 years) [12]. Even after single meals containing 75% of the individual daily Phe allowance an increase of mean plasma Phe concentration of only 13% was observed. Extreme peak concentrations of Phe were not detected and the rises were only transient with Phe levels returning to baseline values within two to three hours. Similarly, MacDonald et al. who monitored Phe concentrations in 16 PKU patients (1 to 18 years) over a 24-hour period found no rise in Phe levels in response to Phe consumption [11].

In a recent study, van Rijn et al. investigated the effect of an additional Phe load on blood Phe concentrations in six adult patients [24]. In this study population Phe concentrations before the study were within the target range of 120–600 μmol/l. Phe loads equivalent to 100% and 200% of each patient’s individual daily Phe intake were given once per week and Phe concentrations in dried blood spots were measured in daily intervals. Mean Phe concentration during the days before the Phe load did not differ significantly from days after the load suggesting that an extra, incidental intake of 100% - and in some cases 200%- of the individual daily Phe intake is tolerated by patients with well-controlled PKU.

Twenty- four- hour variability in blood Phe concentration could also be affected by the amount and distribution of Phe-free amino acid supplement. MacDonald et al. found strong negative correlation between the amount of protein substitute taken by the time of the evening meal and the change in plasma Phe concentrations during the day. The more protein substitute was taken early in the day, the greater was the fall in plasma Phe concentrations during the course of that day [10]. In all of our study patients taking relevant amounts of amino acid supplements the doses were distributed evenly throughout the day. Therefore, we can draw no conclusions on the effects of an uneven distribution on the variability in blood phenylalanine concentrations.

### Short-term effects of exercise on Phe and Tyr concentrations

Our short-term endurance training (ergometry) showed no significant effect on mean Phe concentration within the two hours after the exercise. In contrast, the mean Tyr concentration showed a slight, albeit significant increase within one hour and a sharp decrease during the following hour. To our knowledge, no data are available with respect to the short-term effects of exercise on amino acid concentrations in PKU patients so far. It has been shown that in healthy individuals exercise may have a profound acute effect on protein metabolism [25]: Although protein is not normally an important energy source for exercising muscle, there is a significant increase in the rate of amino acid catabolism during exercise [26]. Furthermore, an increase of whole body protein breakdown has been documented in several studies [27-29]. In the post-exercise state, however, whole body protein synthesis occurs [30], [25]. Phe and Tyr can neither be synthesized nor degraded by skeletal muscle and thus provide a measure of the net rate of protein degradation (i.e. the rate of protein degradation minus the rate of protein synthesis) [26]. An increase in plasma concentrations of both amino acids between 20–90% during exercise has been reported in some studies [31-33] while other authors found Phe and Tyr concentrations to remain stable [30].

As Phe released by protein breakdown cannot be metabolized normally in patients with PKU, more pronounced effects could be expected compared to healthy persons. However, the fact that we have observed very stable Phe concentrations during and after exercise implicates that short-term sportive activity and the concomitant protein breakdown do not put adult patients at risk for extreme peak concentrations of Phe.

### Comparison of amino acid concentrations in serum and dried blood

Amino acid analysis in dried blood spots by tandem mass spectrometry yielded lower concentrations (-28%) compared to the corresponding serum levels. It was tried to reduce common errors in the application of whole blood on filter paper cards by the fact that all dried blood samples were prepared by the same person. The concentration difference between serum and dried blood can be explained by the volume displacement effect [34] in samples containing cellular matter. Deproteinization and centrifugation of serum eliminates the volume fraction of cellular components and distributes the remaining soluble analytes in a smaller volume, thus resulting in a higher concentration value [35]. Because amino acid analysis in dried blood spots is commonly used in the long-term monitoring and dietetic management of PKU patients, it has to be noted that corresponding Phe concentrations in serum are significantly higher.

Notably, we found an unexpectedly poor correlation between Phe concentrations in serum and dried blood spots, whereas Tyr concentrations correlated to a higher degree. As the same analytical methods and samples were used for both amino acids, this lack of correlation is probably not due to preanalytical or analytical differences. We hypothesize that due to stability of Phe concentrations with fluctuations of only 3%, subtle changes in Phe concentration may have been masked by analytical errors resulting in the poor correlation of the two methods. More pronounced changes in blood Tyr concentration led to better statistical correlation.

### Comparison of amino acid concentrations in serum and MD fluid

In our patients the mean Phe concentration measured in MD fluid reached only approximately 60% of the concentration measured in serum. Rolinski et al. studied the determination of amino acid concentrations by subcutaneous MD in nine newborn infants [16]. In contrast to our results, they found higher concentrations of Phe and Tyr in MD fluid compared to plasma (108% and 127% of the corresponding plasma concentration, respectively). Thus, it was concluded that these amino acids are synthesized and/ or released by the subcutaneous tissue [16]. However, it has to be taken into account that tissue composition and metabolic activity of the subcutis in newborns may differ considerably from those in adults [16]. Furthermore, Rolinski et al. studied newborns with hypoglycemia. Since this may prompt proteolysis in order to enable gluconeogenesis, it can be hypothesized that protein catabolism may have contributed to higher tissue amino acid concentrations in the newborns. Independent of the patients’ age the position of the catheter can have a major impact on the relative recovery because vascularisation of the tissue influences the diffusion rate. The position of the catheter in the deep adipose tissue with low metabolic activity would therefore be associated with lower amino acid concentrations in the MD fluid.

We found no significant correlation between Phe concentrations in MD fluid and serum. As already discussed above, the low variability of Phe concentrations could be a complicating factor impeding good correlation results. On the other hand, Rolinski et al. have shown that amino acid values in MD fluid only partly reflect plasma values [16] and suspected a tissue specific amino acid pattern in subcutaneous tissue. As we have only determined Phe and Tyr concentrations instead of a more comprehensive amino acid pattern, we can neither confirm, nor disprove their hypothesis.

Assuming a possible delay in reaching an equilibrium between amino acid concentrations in serum and adipose tissue, we additionally performed correlation analysis with a delay of one and two hours. Such a time lag is likely because the MD samples were collected continuously over a period of one hour, while blood was drawn at the end of this one-hour period. However, even with these time delays no significant correlation could be found.

In summary, subcutaneous MD does not reflect blood Phe and Tyr concentrations in adults with PKU.

## Conclusion

As Phe concentrations show only very slight fluctuations over the day, a single blood sample seems to reflect metabolic control in adult PKU patients in a reliable manner. Most patients have their maximum diurnal Phe and minimum Tyr concentrations in the morning. Microdialysis is not advantageous for monitoring Phe and Tyr levels in adult patients with PKU.

## Competing interests

The authors declare that they have no competing interests. The sponsor had no influence on the content of this study and the interpretation of the results.

## Authors’ contribution

SCG was involved in the planning of the study and data interpretation and drafted the manuscript together with CMB. CMB organised and realised the study visits and analysed the data. She drafted the manuscript together with SCG. AK carried out physical examinations of all patients prior to the inclusion in the study and critically revised the manuscript. H-WC provided technical support with respect to microdialysis. RR was responsible for the statistical analysis of the data. CF and KH provided the location and logistics for the study visits and critically revised the manuscript. JOS was responsible for the laboratory analyses and involved in data interpretation. He also critically revised the manuscript. KOS designed and supervised the study, participated in data interpretation and critically revised the manuscript. All authors read and approved the final manuscript.
